# Sequential boost of intensity‐modulated radiotherapy with chemotherapy for inoperable esophageal squamous cell carcinoma: A prospective phase II study

**DOI:** 10.1002/cam4.2933

**Published:** 2020-02-26

**Authors:** Xing‐Wen Fan, Hong‐Bing Wang, Jing‐Fang Mao, Ling Li, Kai‐Liang Wu

**Affiliations:** ^1^ Department of Radiation Oncology Fudan University Shanghai Cancer Center Shanghai China; ^2^ Department of Oncology Shanghai Medical College Fudan University Shanghai China

**Keywords:** chemoradiotherapy, esophageal squamous cell carcinoma, intensity‐modulated radiotherapy

## Abstract

**Purpose:**

This prospective phase II study aimed to determine the efficacy and tolerability of sequential boost of intensity‐modulated radiation therapy (IMRT) with chemotherapy for patients with inoperable esophageal squamous cell carcinoma (ESCC).

**Methods:**

Patients with histologically or cytologically proven inoperable ESCC were enrolled in this study (ChiCTR‐OIC‐17010485). A larger target volume for subclinical lesion was irradiated with 50 Gy, and then, a smaller target volume only including gross tumor was boosted to 66 Gy. The fraction dose was 2 Gy, and no elective node was irradiated. Concurrent and consolidation chemotherapy of fluorouracil (600 mg/m^2^, days 1‐3) plus cisplatin (25 mg/m^2^, days 1‐3) was administered every 4 weeks, for 4 cycles in total. The primary endpoint was 2‐year progression‐free survival (PFS).

**Results:**

Eighty‐eight patients were enrolled in this study. The median age was 65 years (range: 45‐75 years), and 69 patients (78.4%) were men. With the median follow‐up of 26 (range: 3‐95) months, the 2‐ and 5‐year PFS were 39.3% and 36.9%, respectively, and overall survival (OS) were 57.1% and 39.2%, respectively. Tumor stage and concurrent chemotherapy were independent OS predictors. Major acute adverse events were myelosuppression and esophagitis, most of which were grades 1‐2. Nine percent and 2.3% of patients had grade 3 acute esophagitis and late esophageal strictures, respectively.

**Conclusions:**

Sequential boost to 66 Gy by IMRT with chemotherapy was safe and effective for inoperable ESCC. A randomized phase III study to compare with standard dose of 50 Gy is warranted.

## Graphical abstract

Gross tumor is the main pattern of failure for esophageal cancer received chemoradiotherapy. Sequential boost radiation for gross tumor by IMRT is effective and safe for esophageal cancer, especially for patients who received concurrent chemotherapy.

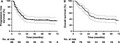

## 1 INTRODUCTION

Esophageal cancer is the seventh most common cancer and the sixth leading cause of cancer‐related mortality worldwide.^1^ Esophageal squamous cell carcinomas (ESCC) comprise the majority of esophageal cancers, with high rates of locoregional recurrence and poor survival.^2^ ESCC often presents at an advanced stage at the time of diagnosis, and most patients with ESCC receive nonsurgical treatments.^3^

Definitive concurrent chemoradiotherapy with a total dose of 50 Gy is the standard nonsurgical treatment for patients with inoperable ESCC, and cisplatin with 5‐fluorouracil (5‐FU) is the most common concurrent chemotherapy regimen.^4^ This protocol was recommended by the Radiation Therapy Oncology Group (RTOG) 8501 study.^5^, ^6^ The irradiation dose considerations were based mainly on the results of the RTOG 94‐05 trial, in which the radiation doses of 50.4 or 64.8 Gy delivered by two‐dimensional radiation techniques were compared, and no significant differences in either locoregional control or other endpoints including quality of life were observed.^7^, ^8^ James et al analyzed the failure patterns in patients with esophageal carcinoma who received definitive chemoradiotherapy, and found that 50% of patients had experienced local failure mostly in the first two years, among which 90% were within the gross tumor volume (GTV), suggesting that the irradiation doses delivered to GTV were probably inadequate.^9^

We used the sequential boost for GTV to 66 Gy with three‐dimensional conformal radiotherapy (3DCRT) without chemotherapy for local advanced ESCC in our previous study.^10^, ^11^ Although the 10‐ year overall survival (OS) rate of 26.6% was promising, the local‐regional recurrence rate of 61.7% remained unsatisfactory. Intensity‐modulated radiotherapy (IMRT) provides superior target volume coverage and conformality with lower doses to normal structures than those associated with 3DCRT.^12^ Steven et al found that ESCC patients treated by IMRT had fewer noncancer‐related deaths, better local‐regional control, and better OS than those treated by 3DCRT.^13^ We hypothesized that sequential boost of IMRT with chemotherapy could increase local control and progression free survival. Therefore, we conducted this prospective phase II study to determine the efficacy and tolerability of sequential boost of IMRT with chemotherapy for patients with inoperable ESCC.

## 2 MATERIALS AND METHODS

### 2.1 Patients

This open‐label, single‐arm, phase II trial was conducted at the Fudan University Shanghai Cancer Center in China. Patients with histologically or cytologically proven ESCC of clinical stages II–IVB (Union for International Cancer Control TNM cancer staging, 6th edition, 2002, nonhematological metastasis),^14^ inoperable disease were eligible for the trial. Patients aged 18‐75 years, with an Eastern Cooperative Oncology Group performance status of 0‐2, and with adequate function of the bone marrow (leukocyte count of 4000/mL and platelet count of 1 00 000/mL), liver (serum bilirubin level of <1.5 mg/dL), and kidneys (creatinine clearance of >65 mL/min) were enrolled. We excluded patients who were pregnant, had previously undergone antitumor treatment, had distant organ metastases, or had a past or present history of other malignancies.

The study protocol was approved by the ethics committee of the Fudan University Shanghai Cancer Center. The– study was conducted in accordance with the principles of the Declaration of Helsinki. All patients provided written informed consent. This clinical trial is registered in the Chinese Clinical Trial Registry (ChiCTR‐OIC‐17010485).

### 2.2 Pretreatment evaluations

Pretreatment evaluations included complete history, physical examination, diagnostic imaging, and laboratory tests. All patients underwent computed tomography (CT) scans of the neck, chest, and upper abdomen, barium esophagram, electrocardiography, and lung function evaluation. Esophageal endoscopy and endoscopic ultrasound (EUS) were also performed. Additional diagnostic tests were performed if there were signs of distant metastases. Positron emission tomography CT was optional but was strongly encouraged.

### 2.3 Radiotherapy

All patients underwent CT‐based treatment simulation with intravenous contrast in the supine position, and images of the neck, thorax, and upper abdomen were obtained with 5‐mm slice thickness.

The GTV referred to the gross tumor volume of the primary esophageal cancer (GTV‐P) and involved lymph nodes (GTV‐N), on the basis of radiologic and endoscopic findings; elective nodal irradiation (ENI) was not employed. Two PTVs were defined. PTV1 was defined as an expansion of 1.2‐to‐1.5‐cm and 1 cm around the GTV‐P and GTV‐N, respectively, and a 3‐cm superior‐inferior expansion of the GTV‐P; the PTV2 was generated using a uniform 0.7‐cm expansion around the borders of the GTV‐P or GTV‐N. The prescription doses for PTV1 were 50 Gy delivered in 2 Gy per fraction per day and 5 fractions per week, starting on the first day of the first chemotherapy cycle. After PTV1 irradiation was completed, PTV2 subsequently received a boost of 16 Gy delivered in doses of 2 Gy per fraction per day and 5 fractions per week, to a total dose of 66 Gy.

An IMRT technique was used, and treatment plans were generated using the Pinnacle treatment planning system (Philips Medical Systems, Pinnacle). Treatments were delivered using 6‐MV photons. The goals were to deliver the prescription dose to at least 95% of the PTV, and 95% of the prescribed dose to at least 99% of the PTV. The dose constraints allowed a maximum dose of <45 Gy to the spinal cord, and a dose not exceeding 20 Gy to 30% of the lung volume (volume of the two lungs minus the PTV) (V20 ≤ 30%). For the heart, a mean dose of <30 Gy was allowed. Tissue inhomogeneity corrections were applied to all dose calculations. Cone beam CT or kV imaging was performed on the first day of radiotherapy to verify the tumor position.

### 2.4 Chemotherapy

Concurrent chemotherapy consisted of two cycles of continuous 5‐FU (600 mg/m^2^/day) and a 1‐hour intravenous infusion of cisplatin (25 mg/m^2^/day) on days 1‐3 and 29‐31. Consolidation chemotherapy was administered for 2 cycles after the completion of concurrent CRT using the same regimen. If the neutrophil and platelet counts were <1.5 × 10^9^/L and <100×10^9^/L, chemotherapy was postponed until the levels returned to normal. During each cycle, chemotherapy was administered only if the nonhematologic toxicities except for hair loss, nausea, and vomiting recovered from >grade 2 to <grade 2; in all such cases, the chemotherapy dose for the subsequent course was reduced by 20%.

### 2.5 Assessments

Toxicity was assessed on a weekly basis during treatment. The first follow‐up evaluation was performed 1 month after the completion of all treatments. Subsequent evaluations were performed every 3 months for the first year, every 6 months for the next 2 years, and yearly thereafter. Physical examination, blood tests, barium esophagrams, CT scans of the neck and chest, upper abdominal CT scans or ultrasound examinations, and ECG were performed at every follow‐up. All acute and late radiation toxicities were evaluated using the RTOG acute and late radiation morbidity scoring criteria and scale, respectively.^15^ All hematological and other nonradiation‐related toxicities were scored using the Common Terminology Criteria for Adverse Events version 3.0 of the National Cancer Institute.

### 2.6 Statistical analysis

The primary endpoint was progression‐free survival (PFS). The secondary endpoints were overall survival (OS), toxicity, and objective response rate (ORR). PFS was measured from the start of treatment until progression or death. OS was measured from the start of treatment until any cause of death. The OS and PFS were calculated using the Kaplan‐Meier method.

The sample size calculations were based on the primary outcome measure of PFS. The 2‐year PFS was expected to improve to 38% from 26% in historic controls.^6^, ^7^ Assuming a type I error (α) of 0.1 and power (1‐ β) of 0.2, and a shedding rate of 10%, we estimated that a total of 88 patients would be required. Univariate and multivariate Cox proportional hazards regression analyses were carried out to assess the significance of variables associated with survival. *P*‐value <.05 was considered statistically significant. Statistical analysis was performed using SPSS (version 17.0, SPSS Inc).

## 3 RESULTS

### 3.1 Clinical characteristics

A total of 88 patients were enrolled between 1 April 2010 and 1 July 2016. Patient characteristics are summarized in Table 1. The median age was 65 years (range: 45‐75 years); 69 patients (78.4%) were men. As per the TNM staging system (UICC, 2002), 22 (25.0%), 36 (40.9%), 16 (18.2%), and 14 (15.9%) patients had stage II, III, IVA, and IVB (lymph node metastasis) tumors, respectively. All primary tumors were located in the thorax.

**Table 1:** The clinical characteristics of 88 patients with esophageal carcinoma

Characteristic	Patients	%
Median age (years)	65 (range 45‐75)	
Gender		
Male	69	78.4
Female	19	21.6
Weight loss before treatment		
None	40	45.5
<5%	30	34.1
≥5%	18	20.4
ECOG Performance status		
0	25	28.4
1	49	55.7
2	14	15.9
Tumor location		
Cervical	0	0
Upper thoracic	31	35.2
Middle thoracic	26	29.5
Lower thoracic	31	35.2
Stage*		
II	22	25.0
III	36	40.9
IVA	16	18.2
IVB (lymph node metastasis)	14	15.9
Tumor length		
<5 cm	54	61.4
5‐10 cm	32	36.4
>10	2	2.2

* Union for International Cancer Control [UICC] TNM cancer staging, 6th edition, 2002.

Treatment characteristics are displayed in Table S1. All patients received radiotherapy ≥50 Gy, and 15 (17.0%) patients did not completed radiotherapy of 66 Gy as scheduled, 5 (5.7%) of which were due to normal tissue constraints during the radiation treatment planning period, and 10 (11.4%) were due to acute toxicity or performance status. Forty‐four (50.0%) patients finished 4 cycles of chemotherapy, 18 (20.5%) patients finished 3 cycles, 12 (13.6%) patients finished 2 cycles, and 14 (15.9) patients finished 1 cycle. Nineteen (21.6%) patients received 2 cycles and 28 (31.8%) patients received 1 cycle of concurrent chemotherapy. Sixteen (18.2%) patients completed radiation of 66 Gy and 2 cycles of concurrent chemotherapy, and 25 (28.4%) patients completed radiation of 66 Gy and 1 cycle of concurrent chemotherapy. Fifty‐eight (65.9%) patients had received chemotherapy before radiotherapy, and the median duration between the first chemotherapy session and radiotherapy was 7 days (range: 0‐37 days).

### 3.2 Survival

**Figure 1:**
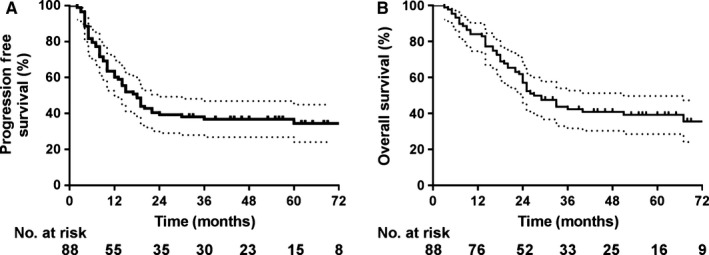
The survival of 88 esophageal cancer patients. (A) progression‐free survival. (B) overall survival

At the final follow‐up on March 2019, the median follow‐up duration was 26 months (range: 3‐95 months). The median PFS was 18.0 months (95% confidence interval [CI]: 13.4‐22.5 months), and the 1‐year, 2‐year, and 5‐year PFS rates were 60.2%, 39.3%, and 36.9%, respectively (Figure 1A). The median OS was 27.0 (95% CI: 19.8‐34.1) months, and the 1‐year, 2‐year, and 5‐year OS rates were 84.1%, 57.1%, and 39.2%, respectively (Figure 1B).

**Figure 2:**
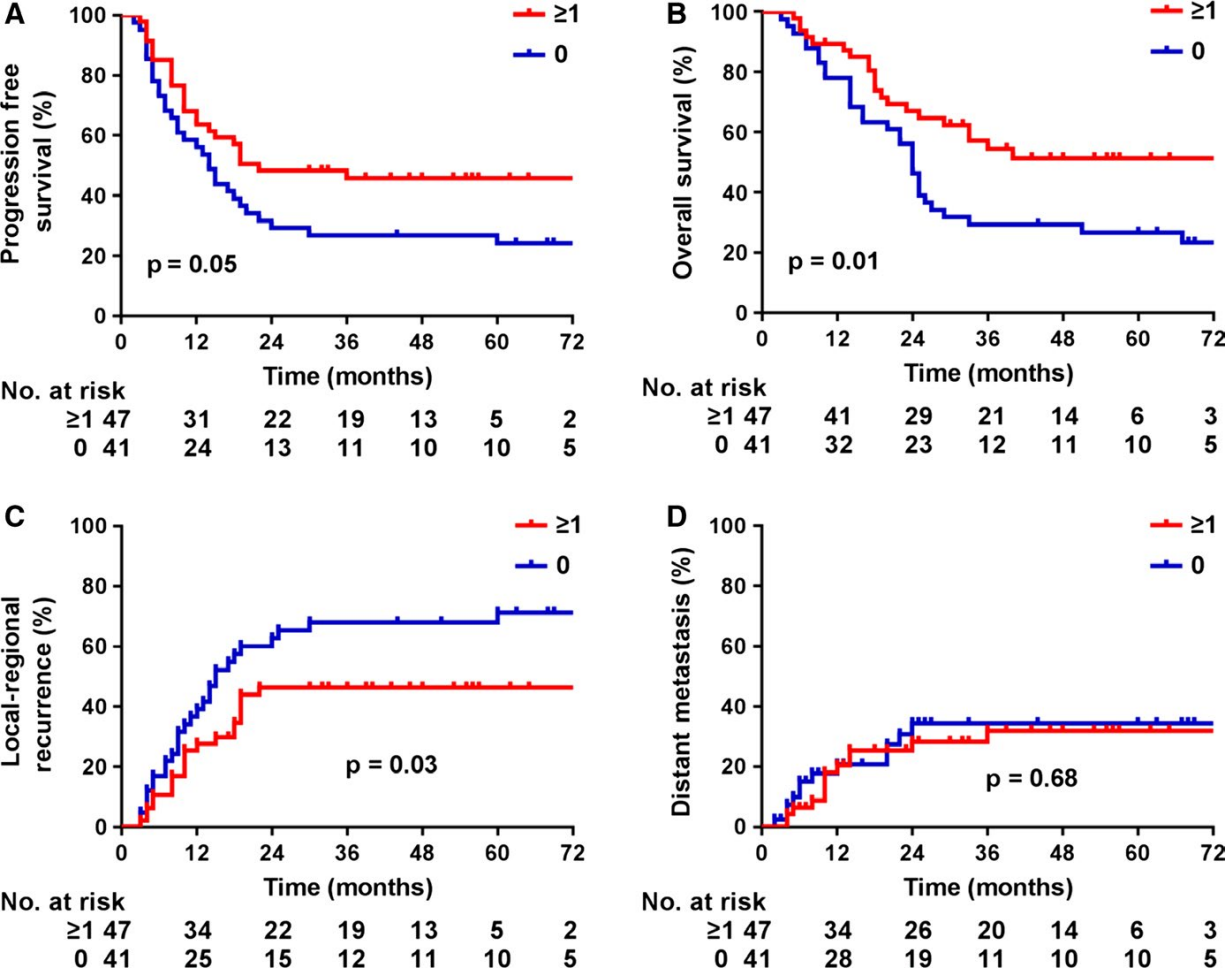
Survival and relapse according to concurrent chemotherapy. (A) progression‐free survival. (B) overall survival. (C) local‐regional recurrence. (D) distant metastasis

Univariate Cox regression analysis indicated that patients with earlier tumor stages, higher radiation doses, or at least one cycle of concurrent chemotherapy survived longer. Multivariate Cox regression analysis indicated that tumor stage (hazard ratio: 2.32, 95% CI: 1.09‐4.94, *P* = .03) and concurrent chemotherapy (hazard ratio: 0.56, 95% CI: 0.32‐0.99, *P* = .05) were independent OS predictors (Table 2). The median PFSs for patients who received at least one cycle of concurrent chemotherapy and no concurrent chemotherapy were 22 months and 14 months, respectively; the 2‐year PFSs were 48.3% and 29.3%, respectively; and the 5‐year PFSs were 45.8% and 24.1%, respectively (log‐rank, *P* = .05, Figure 2A). The median OSs for patients who received at least one cycle of concurrent chemotherapy and no concurrent chemotherapy were not reached and 24 months, respectively; the 2‐year OSs were 64.7% and 46.3%, respectively; the 5‐year OSs were 51.4% and 26.6%, respectively (log‐rank, *P* = .01, Figure 2B). Patients who received at least concurrent chemotherapy had lower local‐regional recurrences (log‐rank, *P* = .03, Figure 2C), but there was no significant impact for distant metastasis (log‐rank, *P* = .68, Figure 2D).

**Table 2:** Cox regression analysis of the morality risk in patients with esophageal cancer

Variable	Univariate analysis			Multivariate analysis		
	HR	95% CI	*P* value	HR	95% CI	*P* value
Gender						
Female vs Male	1.72	(0.84‐3.54)	.14			
Age						
<65 vs ≥ 65	1.43	(0.83‐2.47)	.20			
Stage						
III/IV vs II	2.33	(1.10‐4.96)	.03	2.32	(1.09‐4.94)	.03
Length						
≤5 cm vs > 5 cm	1.42	(0.82‐2.46)	.21			
Radiation dose						
66 Gy vs < 66 Gy	0.47	(0.24‐0.90)	.02	0.55	(0.28‐1.07)	.08
Concurrent chemo						
≥1 vs 0	0.51	(0.29‐0.89)	.02	0.56	(0.32‐0.99)	.05

### 3.3 Patterns of failure

**Figure 3:**
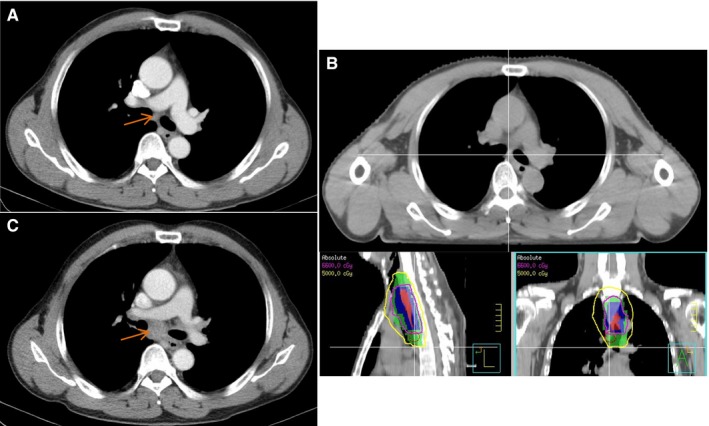
One example for lymph node recurrence outside the PTV. (A) pretreatment, the short diameter of subcarinal lymph node (orange arrow) was 6 mm. (B) the radiotherapy target and radiation dose coverage. The subcarinal lymph node was out of plan tumor volume (PTV). Red area, gross tumor volume; blue area, PTV‐2; green area, PTV‐1; purple line, radiation dose of 66 Gy; yellow line, radiation dose of 50 Gy. (C) the volume of subcarinal lymph node (orange arrow) increased significantly 3 months after radiotherapy

Among 56 patients with progression, 49 (55.7%) were local‐regional recurrences, 25 (28.4%) were distant metastases, and 18 (20.5%) were both. The 1‐year, 2‐year, and 5‐year local‐regional recurrence rates were 33.1%, 53.9%, and 59.4%, respectively. The 1‐year, 2‐year, and 5‐year distant metastasis rates were 20.8%, 31.2%, and 33.4%, respectively. For local‐regional recurrence, 34 (38.6%) were primary tumor recurrences, 21 (23.9%) were lymph node recurrences, and 6 (6.8%) were both. The region of lymph node recurrences included: upper supraclavicular, 11 (12.5%); mediastinal, 8 (9.1%); hilar, 4 (4.5%); abdominal, 5 (5.7%). Five (23.8%) were in the GTV, 2 (9.5%) were out of GTV but in the PTV, and 14 (66.7%) were out of PTV. All lymph node failures out of PTV could be found in the CT when diagnosed, in spite of small diameter. One example is shown in Figure 3. Twelve patients (64.3%) developed both lymph node recurrence and distant metastasis. The pattern of metastasis was as follows: lung, 21 (23.9%); liver, 4 (4.5%); bone, 4 (4.5%); plural, 3 (2.3%), and spleen, 1 (1.1%).

### 3.4 Treatment‐related toxicity

The treatment‐related toxicities are summarized in Table 3. The major adverse events were related to myelosuppression and esophagitis. The incidence rates of leukopenia of grades 1‐2 and 3 were 65.9% and 14.8%, respectively. None of the patients experienced grade 3/4 neutropenia with fever. Patients with acute radiation esophagitis of grades 1‐2 and grade 3 occurred in 60.2% and 9.0%, respectively. Acute radiation pneumonitis of grades 1‐2 and grade 3 occurred in 19.3% and 2.3% of patients, respectively.

**Table 3:** Adverse events during treatment and follow‐up (CTCAE version 3.0)

Toxicity	Grade 1 or 2 n (%)	Grade 3 n (%)
Leukopenia	58 (65.9)	13 (14.8)
Neutropenia	47 (53.4)	11 (12.5)
Anemia	60 (68.2)	7 (8.0)
Thrombocytopenia	30 (34.1)	9 (10.2)
Creatinine	2 (2.2)	0 (0)
Vomiting & nausea	21 (23.9)	13 (14.8)
Anorexia	35 (39.8)	18(20.5)
Diarrhea	2 (2.2)	0(0)
Febrile neutropenia	0 (0)	0 (0)
Pneumonitis*	17 (19.3)	2 (2.3)
Esophagitis*	53 (60.2)	8 (9.0)
Pulmonary fibrosis*	7 (5.7)	0 (0)
Esophageal stricture*	2 (2.3)	2 (2.3)

Abbreviation: CTCAE = Common Terminology Criteria for Adverse Events.

* RTOG Acute Radiation Morbidity Scoring Criteria and RTOG Late Radiation Morbidity Scoring Scale.

Late toxicity was noted in 9 patients (7.9%) who were followed up for more than 3 months after CRT. Seven patients (5.7%) experienced grade 2 radiation pulmonary fibrosis. Two patients (2.3%) developed moderate esophageal strictures and were placed on semi‐liquid or liquid diets. Severe esophageal strictures occurred in 2 patients (2.3%) that improved with placement of esophageal stents.

## 4 DISCUSSION

This is the first phase II prospective study to determine the safety and efficacy of sequential boost of IMRT with chemotherapy for patients with inoperable ESCC. Two‐year PFS, the primary endpoint, was 39.3% for all recruited patients in this study, reaching the preset endpoint. The 5‐year OS was 39.2% for all patients, and was 51.4% for those who received at least one cycle of concurrent chemotherapy. The survival results of this study were promising. A randomized phase III study to compare sequential boost to 66 Gy with standard dose of 50 Gy is warranted in the future.

Local‐regional recurrence was the primary failure pattern, accounting for 55.6% of all patients, and 87.5% of those with progression. The local failure rate of this study was similar to that of radiation dose of 50.4 Gy, which was 42‐54%,^6^, ^9^, ^16^, ^17^ appearing not to have improved with increased radiation dose with sequential boost method. However, the PFS was improved compared with historical controls.^18^ This may be because the distant metastasis rate was low (28.4%), and most distant metastases (72.0%) were combined with local‐regional recurrences. Another radiation dose escalation method, simultaneous integrated boost (SIB), has also been explored^17^, ^19^, ^20^, ^21^, ^22^, ^23^, ^24^, ^25^, ^26^; local control of 67.5%‐78.6% was reported.^17^, ^20^, ^22^ However, local control of ESCC was not improved by SIB, compared with standard dose.^17^, ^26^ These findings suggest that improvement of local‐regional control by radiation dose escalation remains a matter of controversy.

Local‐regional control in this study was better than that of our previous phase II study, in which patients were treated with same radiation dose by three‐dimensional radiotherapy without chemotherapy; the local‐regional recurrence rate was 61.7%.^11^ Patients receiving at least 1 cycle of concurrent chemotherapy had better local‐regional control than those without concurrent chemotherapy (53.6.9% vs 28.7%, *P* = .03), suggesting the indispensable role of concurrent chemotherapy, which was demonstrated by RTOG 8501 in the 1990s.^5^, ^6^ There are several reasons for low concurrent use of chemotherapy in this study. First, most (65.9%) of the patients received their first cycle of chemotherapy before radiotherapy because the radiation equipment in our department was frequently in use and there were long wait times. Patients refused concurrent chemotherapy because fear of discomfort associated with chemotherapy. Another reason was that 60%‐85% of ESCC patients were malnourished,^27^ possibly decreasing tolerance to chemotherapy. Enteral nutrition may increase the completion of chemoradiotherapy (96% vs 89%, *P* = .03, NCT02399306). Decreasing the drug dose and increasing the frequent could decrease the toxicity with no impact to efficacy of chemotherapy.^28^ Therefore, in the future, we will try the weekly concurrent chemotherapy protocol and enteral nutrition for patients.

Involved field irradiation (IFI) was used in this study, and more out‐field failures (15.9%) were found compared in another study that used elective nodal irradiation (ENI) (9.3%).^16^ In another phase II trial to study the IFI, in which 18‐fluorodeoxyglucose positron‐emission tomography was used, only 2/58 patients experienced out‐field failure.^29^, ^30^ In the present trial, no positron‐emission tomography was used. However, all lymph nodes of recurrence could be found in their diagnostic CT in spite of small diameter, suggesting that the criteria of positive lymph nodes should be expanded, especially when nodes are in high‐risk regions. Furthermore, 64.3% of lymph node recurrences were combined with distant metastasis. Therefore, we believe that it would be sufficient to irradiate all nodes in the high‐risk regions, but not prophylactically in all high risk‐regions.

The major acute adverse events in this study were myelosuppression and esophagitis, most of which were grades 1‐2. Grade 3 esophagitis occurred in 9.0% patients, similar with previous reports with radiotherapy of 50 Gy.^18^, ^31^, ^32^ A total of 2.3% of patients had grade 3 late esophageal strictures, which was 4‐5% in SIB studies.^20^, ^22^ All patients finished radiotherapy of 50 Gy, and 10 (11.4%) patients terminated their treatment during the boost period due to acute toxicity or performance status. The radiotherapy completion was not as good as the SIB strategy^20^, ^22^; the reason may be explained by the shorter fractions of SIB, and all the termination of radiation occurred in the final 8 fractions.

One limitation of this study is the low treatment completion. Fifteen (17.0%) patients did not complete radiotherapy of 66 Gy, and 41 (46.6%) patients did not receive concurrent chemotherapy. Patients refused the concurrent chemotherapy mainly because of fear of the toxicity of chemotherapy, especially digestive discomfort. Nevertheless, we found that concurrent chemotherapy significantly affected survival. Despite the fact that we adjusted for stage, age, gender, location, and length of tumor, the group patients without concurrent chemotherapy may include those with potential poor prognostic factors such as malnutrition. Patient education and management should be strengthened to assure treatment completion in future studies.

## 5 CONCLUSIONS

Sequential boost to 66 Gy by IMRT with chemotherapy is safe and effective for patients with inoperable ESCC. The 5‐year OS of 39.2% for all patients and 51.4% for those who received at least one cycle of concurrent chemotherapy were promising. A randomized phase III trial to compare sequential boost to 66 Gy by IMRT with standard dose of 50.4 Gy is warranted in the future.

## CONFLICT OF INTEREST

No authors declare conflicts of interest.

## AUTHOR CONTRIBUTIONS

Kai‐liang Wu and Xing‐wen Fan designed the study. Xing‐wen Fan and Hong‐bing Wang drafted the manuscript. Hong‐bing Wang, Jing‐Fang Mao, and Ling Li contributed the materials, and interpreted the data. All the authors read and approved the final manuscript.

## Data Availability

The data will be provided upon the request.
