# Supplementary material for: Sequential boost of intensity‐modulated radiotherapy with chemotherapy for inoperable esophageal squamous cell carcinoma: A prospective phase II study
Source: Cancer Med. 2020 Feb 26;9(8):2812–9. doi: 10.1002/cam4.2933 (PMC7163105; doi:10.1002/cam4.2933)
Supplement: Supplementary file 1 [file CAM4-9-2812-s001.docx]

eTable 1. Treatment characteristics.

| Characteristics | No. (%) |
| --- | --- |
| Radiation Dose  66 Gy  60-65 Gy  50-60 Gy | 73 (83.0)  6 (6.8)  9 (10.2) |
| Total chemotherapy cycles  4 cycles  3 cycles  2 cycles  1 cycle | 44 (50.0)  18 (20.5)  12 (13.6)  14 (15.9) |
| Concurrent chemotherapy cycles  2 cycles  1 cycle  0 cycles | 19 (21.6)  28 (31.8)  41 (46.6) |
| Chemotherapy cycles before radiotherapy  1 cycle  0 cycles | 58 (65.9)  30 (34.1) |
| Days from chemotherapy to radiotherapy  Median (range) | 7 (0-37) |
| Chemotherapy delayed^1^  2^nd^ cycle  3^rd^ cycle  4^th^ cycle | 61 (68.9)  71 (80.7)  77 (87.5) |

^1^chemotherapy delayed more than 7 days or termination.
